# Assessing experts’ perspectives on challenges in substance misuse prevention, harm reduction, and treatment to shape funding priorities in New York State

**DOI:** 10.1186/s12954-024-01045-3

**Published:** 2024-07-15

**Authors:** Daniel J. Kruger, Hilary M. Kirk, Kenneth E. Leonard, Joshua J. Lynch, Nancy Nielsen, R. Lorraine Collins, Joseph W. Ditre, Debbian Fletcher-Blake, Susan A. Green, Aaron Hogue, Julia K. Hunter, John M. Marraffa, Brian M. Clemency

**Affiliations:** 1 Department of Emergency Medicine, Jacobs School of Medicine and Biomedical Sciences, University at Buffalo, University at Buffalo Gateway Building, Suite 420, 77 Goodell St., Buffalo, NY 14203 USA; 2 Department of Community Health and Health Behavior, School of Public Health and Health Professions, University at Buffalo, 3435 Main Street, Buffalo, NY 14214 USA; 3 Clinical and Research Institute On Addictions, University at Buffalo, 1021 Main St, Buffalo, NY 14203 USA; 4 Department of Psychology, Syracuse University, 430 Huntington Hall, Syracuse, NY 13244 USA; 5 VIP Community Services, 770 E 176Th St, Bronx, NY 10460 USA; 6 School of Social Work, University at Buffalo, 85 Baldy Hall, Buffalo, NY 14260 USA; 7 Partnership to End Addiction, 711 Third Avenue, 5Th Floor, Suite 500, New York, NY 10017 USA; 8 United Health Services Hospitals, Inc., 10-42 Mitchell Ave, Binghamton, NY 13903 USA; 9 Kinney Drugs, 6333 Route 298, Suite 305, Syracuse, NY 13057 USA

**Keywords:** Substance misuse, Prevention, Harm reduction, Treatment, Challenges

## Abstract

**Background:**

Drug overdose is a leading cause of death and opioid-related deaths increased by more than 300% from 2010 to 2020 in New York State. Experts holding a range of senior leadership positions from across New York State were asked to identify the greatest challenges in substance misuse prevention, harm reduction, and treatment continuum of care. Expert input was used to shape funding priorities.

**Method:**

Individual semi-structured interviews of sixteen experts were conducted in April and May 2023. Experts included academics, medical directors, leaders of substance misuse service agencies, administrators of a state agency, a county mental health commissioner, the president of a pharmacy chain, and a senior vice president of an addiction-related national non-profit. Zoom interviews were conducted individually by an experienced qualitative interviewer and were recorded, transcribed, and coded for content. An initial report, with the results of the interviews organized by thematic content, was reviewed by the research team and emailed to the expert interviewees for feedback.

**Results:**

The research team identified five major themes: 1. Siloed and fragmented care delivery systems; 2. Need for a skilled workforce; 3. Attitudes towards addiction (stigma); 4. Limitations in treatment access; and 5. Social and drug related environmental factors. Most experts identified challenges in each major theme; over three-quarters identified issues related to siloed and fragmented systems and the need for a skilled workforce. Each expert mentioned more than one theme, three experts mentioned all five themes and six experts mentioned four themes.

**Conclusions:**

Research, educational, and programmatic agendas should focus on identified topics as a means of improving the lives of patients at risk for or suffering from substance use-related disorders. The results of this project informed funding of pilot interventions designed to address the identified care challenges.

## Introduction

Approximately two million New Yorkers exhibit substance use disorder behaviors [1]. According to the most recent Behavioral Risk Factor Surveillance Survey data, one in five adults in New York State reported,heavy, or binge alcohol drinking in 2022 [2]. The rate of overdose deaths involving an opioid quintupled from 2010 to 2021 with nearly 5,000 people dying of an opioid overdose in 2021 [3]. The overdose death rate for synthetic opioids other than methadone increased from 12 to 18.9 per 100,000 from 2019 to 2020 [4]. Research studies have examined challenges throughout the substance use continuum of care, but few have explored system leadership perspectives, [5, 6]. which may add value given leaders’ unique understanding of the regulatory and practical challenges in overseeing and running systems of care.

The New York State Office of Addiction Services and Supports (OASAS), the University at Buffalo’s Clinical and Research Institute of Addictions (CRIA), and the Medication for Addiction Treatment & Electronic Referrals (MATTERS) Network established a Research Institute to identify gaps in the provision of science-based models within the New York State system of addiction care (prevention, harm reduction, treatment services). The goal of this initiative is to identify the challenges in New York State from the perspective of leaders in various capacities throughout the State and address these challenges through research and funding of pilot projects. Increasing health equity for service recipients was included as a critical element of identifying and addressing these challenges. The current manuscript documents the results as generalizable knowledge that, although centered in New York State, could be useful to other locations. We also provide an exemplar for a knowledge-generation process that other locations could undertake to yield similar rewards for their regions.

## Method

The current project was sponsored by the New York State Office of Addiction Services and Supports (OASAS) to identify priority areas for enhancing the systems of prevention, treatment, and harm reduction in the State. The OASAS/CRIA Research Institute invited individuals in leadership and professional roles related to substance use to join a Clinical and Research Expert Panel. Individuals were invited to participate based on discussions between the Institute leadership team and OASAS. Expertise and extensive experience as a New York State-based practitioner, researcher, or other professional in substance use disorder prevention and treatment, as well as involvement with the OASAS system of services were the criteria for inclusion.

### Participants

The Institute sent email invitations on March 28, 2023, to 21 individuals describing the project, project objectives, and requirements for participants. The email notification offered an incentive of $500 for up to four hours of participation, including interviews and providing feedback on interview summaries. Individuals consented to participate in the study by replying to the email with an indication of their interest. Sixteen experts agreed to participate and were interviewed (76% acceptance rate) in April and May 2023. Five experts were academics (professor, associate professor, or clinical professor), representing clinical psychology, psychiatry, public health, and social work. Four experts were chief executive officers or executive directors of substance use disorder service delivery agencies. Two experts were departmental medical directors, and two experts were OASAS administrators. The other experts included a county mental health commissioner, a president of a large regional pharmacy chain, and a senior vice president of a national non-profit focused on addiction prevention and treatment.

### Data collection

An experienced qualitative researcher conducted interviews individually via Zoom (Zoom Video Communications, San Jose CA), which were recorded and transcribed. Interviews ranged from 30 to 60 min depending on expert availability. Most interviews were conducted by two members of the research team, with some interviews including one or three members of the research team. The qualitative researcher participated in all interviews and two other research team members participated in interviews as available. Interviews were semi-structured with the standard lead question, “What is the greatest challenge in substance abuse prevention, treatment and recovery facing in New York?” and follow-up questions, “How many people does it affect?” (What is the magnitude of the problem?) and “What are the consequences?” (What is the severity of the problem?). Additional questions were based on the content of panelist responses, to clarify and specify challenges.

### Data analyses

The qualitative researcher analyzed interview transcripts and identified themes and subthemes. Themes, subthemes, and content were discussed and finalized by the research team. The team sent an initial report with the results of the interviews organized by thematic content to the expert interviewees for review and feedback. The qualitative researcher calculated the proportion of experts identifying each theme and the number of themes identified by each expert.

## Results

Five themes of major challenges emerged during the interviews (Table 1): 1. Siloed and fragmented care delivery systems; 2. Need for workforce training; 3. Attitudes towards addiction (stigma); 4. Limitations in treatment Experts reported that stigma creates barriers to critical resources, such as acquiring naloxone and medications for substance use disorders. access; and 5. Social and drug related environmental factors. The most common theme was siloed and fragmented systems, including the separate regulatory bodies overseeing substance use, mental health, and medical care with resulting differential funding streams, reimbursement models, service provision standards, data collection, and dissemination protocol. This theme was identified by nearly 90% of the experts. Experts indicated that there needs to be a continuous model of care, with no artificial distinctions between prevention, treatment, and harm reduction; between inpatient and outpatient care; and between substance misuse treatment and mental health treatment. Experts recommended developing consistent programmatic metrics and policy alignment especially among State agencies.

**Table 1:** Representative quotes for themes and subthemes

1. Siloed and fragmented systems (87.5%)
1.1.* Need for continuum of services with no artificial distinctions *(81.3%) • “Transitions in care is an old problem in the system, and none of this is unique to New York and people have talked about hot handoffs, warm handoffs for years, and I’ve tried to throw case management at it. We haven’t really solved the problem.” • “The mental health world and the addiction world came more together versus separate silos…now there are treatment programs that address both at the same time but we’re still antiquated, around who funds what part. You go to an agency here, and they have the mental health side, and they have the addiction side, and they talk. But your primary diagnosis is addiction, or your primary is the mental health diagnosis.” • “I was on a call and so I just found it funny that someone actually said ‘Well, as a counselor, do you treat the mental health issue or the substance use issue first’? And I was like, ‘Yeah, treat it all.’ You first of all make sure that patient is safe, and you don’t care if it’s a mental health or substance.” • “Artificial, but for me, useful distinctions along the continuum of care into prevention, treatment, and recovery. Acknowledging upfront that there’s a lot of overlap and that a lot happens, and transitions and people come into treatment and then go into recovery, go back to treatment, and then you’re preventing things.”
1.2.* Need for no wrong door approach for entry into services *(81.3%) • “We have to create a system in which whatever door you touch lets you in.” • “I think hopefully, we’re creating a no wrong door trajectory” • “We could do a better job … starting people on medications in the hospital setting and linking them to care afterwards”; • “But people don’t really see, don’t understand, if a patient gets admitted and they treat the endocarditis but don’t treat the addiction. This happens all the time in our hospital system.”;
1.3.* Need for unified policies and progress metrics* (62.5%) • “If you look at New York State, things are so segmented, so you’ll have OASAS saying one thing and [?] saying a different thing and doing things differently and requiring different things” • “It’s super difficult to be in alignment with all the three strategies that should be in alignment, I shouldn’t have to find ways for them to align, and I often feel like that’s what I’m doing, or our partners are doing, it just causes more paperwork, less efficiency. It causes issues in terms of over burdening their staff…. There needs to be more cohesiveness with approaches from the higher levels.” • “One of the biggest barriers has been the variety of different systems that are used to collect data and the different data that is collected”
2. Need for a skilled workforce (75.0%)
2.1.* General shortage of highly skilled health professionals *(68.8%) • “The workforce is another big challenge. There are just not enough people to do the work. Providers, counselors, nurses.” • “Right now, we’ve got a crisis with the workforce. We’ve had a shrinking workforce for a long time…There’s low pay, high levels of stress, and people are either removing themselves or not entering those professions.” • “So these younger, hopefuls, when they are doing a good job, might get put in a position that they might not feel qualified for. And there’s not enough time to train them, because there’s such a workforce shortage.” • “Our workforce is not equipped for the requirements that we have of people being able to do this type of work in regards to the complexity of people’s mental health and addiction pieces.”
2.2.* Challenges with training, including frequent staff turnover *(62.5%) • “So it’s more support for those newer counselors, and they often get thrust into large caseloads, so it can cause burnout and early exit out of the field, because the young counselors are overwhelmed”; • “There’s no time to implement a brand-new process and make sure that they can do it with fidelity.” • “We don’t train physicians, nurses, NPS, even social workers, or any kind of other license, marriage and family license, mental health counselors. We don’t train them in addiction. We don’t expose them to feel placements unless they kind of seek it out on their own, or it just kind of happens.” • “You have to start looking at pre-service training being more intensive with these kinds of these kinds of evidence-based practices, we have to start learning them, earlier on in their training and their schooling and their education.”
2.3.* Competition from other sectors with higher wages and better working conditions *(25.0%) • “There’s competition with Starbucks, Tim Hortons, like just entry level jobs in the community, get paid more than perhaps people who have, you know, Master’s degrees in counseling or education, and are doing prevention or recovery work.” • “They were burning out, they left for other things” • “There’s just a horrible crisis, not enough people to hire to do all the work, not even just in human services, there’s not enough people for all the jobs”
3. Attitudes towards addiction/stigma (68.8%)
3.1.* Negative attitudes (stigma) and misconceptions across stakeholders and populations *(50.0%) • “I struggle on a daily basis with the programs that I oversee and the community that I work in, dealing with stigma.” • “Prevention is the first step of education prevention and breaking down barriers of stigma. It’s almost obvious, I think we have to figure that out, but that’s got to run, I think, as an undercurrent, anything else we do in the treatment and recovery worlds.” • “I think that there’s still quite a bit of stigma and morality around substance use and the reasons each person thinks that they might be using” • “How do you get the stigma out of Methadone? I worked in a Methadone clinic. It’s really an effective treatment for a lot of people. But there’s so much stigma related to it that people who need it won’t even try it… medication for substance use disorder needs to be addressed and utilized more.”
3.2.* Lack of understanding that a substance use disorder is a chronic disease requiring long-term care *(50.0%) • “An addiction is a chronic, relapsing, remaining brain disease. And so, people in addiction care should be treated at the primary care level, and if primary care is unable to manage their use disorder, in this case opioids, then they should be referred to…an opioid treatment program.” • “It’s a lack of understanding that this is an illness similar to diabetes or cardiovascular disorders, or anything else.” • “There’s still this is a correlator with opiates, a fundamental misunderstanding of the chronicity. A fundamental misunderstanding of the chronicity of addiction in general. These are patients who should be thought of as long-term care patients.” • “I think that it’s still a big barrier to try and get folks to look at it as a medical kind of condition, or that they are our brain changes, or like the chemistry of addiction in the treatment community, in the community at large.”
3.3.* Stigma can have a racial element and differentially impact minority communities *(25.0%) • “We saw that with regard to prescribing opiates for pain, [someone] who was African American was more likely just drug seeking, and besides, they should be able to withstand that pain because they’re black.” • “[There is a perception that] our black and brown brothers and sisters don’t have the same tolerance of pain at a doctor’s office that you and I would. And I think that’s a huge issue. If I walk in and I tell my doc I’m in pain, I’m gonna get something. But a black patient with the same level of pain walks in, they’re not going to be taken seriously.”
4. Limitations in treatment access (62.5%)
4.1.* Access to treatment *(56.3%) • “The greatest challenge, it’s a couple of things, I think. Definitely access to care.” • “Yes, everybody is going to say that stigma and access are [the greatest challenges]. But how do we change those 2 pieces?” • “Limited access to services, especially in areas with few harm reduction or treatment providers.” • “There was a guy in the cemetery yesterday, 27 years old, that was not doing well. He had just taken meth about 4 h before, and he was on something else and something else. And there was, I would say, caring and direct intervention by law enforcement. I really believe that this person probably definitely doesn’t have the same type of insurance coverage as somebody else might have in terms of them being in a position to get what’s needed.”;
4.2.* Disparities in access to treatment *(50.0%) • “One of the things that’s really concerning is the barriers to getting treatment, and the way and the way that disproportionately affects minorities and underserved individuals.” • “I think that there’s not enough evidence-based practices for communities of color.” • “So we thought, with a certain segment of our population, those that look different than you and I, in terms of those that are brown skinned, or whatever, and so there wasn’t as much quote resource and or I’ll even say aggressiveness to try to fix this, and the whole piece now around the opiate stuff.” • “Buprenorphine is not easily accessible to, you know, based on race in the city…poor communities that are overrepresented with black and Latino individuals tend to not have as much funding or resources available to them, and available workforce, so they tend to have older models of care.”
5. Environmental factors (56.3%)
5.1.* Social determinants of health *(43.8%) • “The other major piece to the puzzle is around how housing and substance use interact… a lot of our folks are unhoused. Don’t have a phone, so it’s really hard to connect with them.” • “With chaotic use, it’s really hard for people to maintain their housing anyway, in terms of income and making sure that they get the documentation that’s needed …it’s hard for folks to stay in the same place throughout their treatment or even if they’ve completed a treatment or recovery program to land in a place that is safe for them.” • “there’s a direct relationship to the number of outlets of whatever, and the amount of use…communities that have lower levels of outlets have lower use rates.” • “We cannot separate what we are calling the social drivers of health. Or I personally call them the impediments, the social impediments to health.”
5.2.* Current drug environment* (31.3%) • “What people were experimenting with 50 years ago didn’t kill them in the same way that the Fentanyl is killing today.” • “I saw Director Milgram from the DEA talking about the criminal side of the fentanyl problem. The image that I had and how the materials coming from China and the cartels in Mexico make the drug, and they’re flooding our community. They’re poisoning our communities. I suddenly had the image of like we’re doing CPR on a patient, and we don’t understand that there’s someone knifing them on the side. We’re trying to save a patient that a lot of people are trying to kill.” • “I think when you look at society today, an opioid is kind of the easy button and I don’t feel well today or I’m in pain, I could take this substance and make it go away. We have to look at prescribing patterns… because a lot of opioid addiction happens accidentally, they didn’t know that they were going to struggle.” • “There’s other dynamics at play with supply that that our services can’t control…the biggest challenge is to mitigate all the supply issues that are impacting demand.”

*Note:* Percentages represent the proportions of experts expressing themes and subthemes

The second most common theme, identified by three-quarters of the experts, was the difficulty maintaining a skilled workforce, including challenges in hiring, training, and retaining qualified individuals. Experts noted that a threat to recruitment and retention in community-based behavioral health settings is wage competition from larger health systems which may also be able to provide desirable working conditions such as smaller caseloads. There is a paucity of training for entry-level positions and a lack of integration of substance use disorder topics into educational curricula for health professions. Moreover, experts noted a high degree of variability in training programs’ programmatic quality and adherence to evidence-based practices. The experts interviewed also expressed a need for interprofessional education so practitioners with different skill sets can work as a team.

As a result of these forces, there is a general shortage of highly skilled addiction medicine professionals across sectors, particularly in communities with the most severe needs and in smaller organizations without academic affiliations to support the hiring of individuals with desired skills. Given a workforce in need of further training in evidence-based practices, it was noted that service providers cannot uniformly take advantage of useful training programs due to financial and time costs. Furthermore, experts noted there is competition from other employment sectors with higher wages and more favorable working conditions, including retail and service industries. This can create a problem in that training staff may paradoxically result in staff leaving, as they gain the skill sets desired for positions with higher compensation. Staff who are trained but remain in the agency are often promoted to higher-level positions which require skills they may not have. Agencies often have suboptimal management structures and may reduce available time for direct client services.

The third most common theme was attitudes towards addiction/stigma, identified by two-thirds of experts. These barriers included properties of the treatment providers, students, educators, patients, and the community. Experts reported that people do not understand that addiction is a chronic disease, and instead generate their own interpretations of the reasons why other people use substances. This is contrary to the experts’ notion (and position of the American Society of Addiction Medicine) that addiction is a chronic condition which may require continual monitoring and/or treatment. The experts believed that many people do not consider a person to be recovered or in recovery until they are no longer taking medications or receiving therapy. Moreover, experts felt that stigma can also have a racial/ethnic element and differentially impact minoritized communities.

The fourth theme, identified by most experts, was limitations in treatment access. System barriers included resistance from hospitals worried about the financial costs of high risk/high need patients, hesitancy to prescribe buprenorphine for people with co-occurring mental health conditions or use of another substance in addition to opioids, and restricted access to services (including buprenorphine and methadone), especially in under-resourced areas. Requirements for methadone treatment may be burdensome (e.g., presenting to the opioid treatment program regularly for methadone dosing). Experts also noted disparities in access to treatments, including evidence-based practices for communities of color.

The fifth theme, identified by most experts, was environmental factors including social determinants of health and conditions in the evolving drug supply. Social determinants of health included lack of stable employment, income, and housing, as well as lack of personal documentation (e.g., valid identification, employment records) that would facilitate access to these resources. Experts noted that there is a continuing supply of illicit drugs and that these drugs are more potent and dangerous today than in the past, particularly fentanyl and xylazine. Some experts remarked that a small proportion of the population will always be using whatever substance is available. Experts emphasized the importance of providing access to harm reduction services and programs to people who use substances.

Table 2 depicts the pattern of subtheme identification by expert. Individual experts are indicated by a numerical code assigned based on the number of subthemes identified. The number of subthemes identified ranged from 11 to 4 out of 13, with an average of 7 subthemes identified by each expert. Three experts mentioned issues associated with all five themes, six experts mentioned issues associated with four themes, five experts mentioned issues associated with three themes, and one expert mentioned issues associated with two themes. The number of experts mentioning each subtheme ranged from 13 to 4 out of 16, with an average of 7 experts per subtheme. The two experts most focused on workforce challenges (15 and 16) were in administrative roles in service delivery organizations. These individuals also identified challenges consistent with other major themes.

**Table 2:** Subthemes of challenges by number of experts expressing themes and number of themes expressed by each expert

Topic	Expert	Total experts															Per subtheme
	1	2	3	4	5	6	7	8	9	10	11	12	13	14	15	16	
1.1	X	X	X	X	X	X	X	X	X	X	X	X		X			13
1.2	X	X	X	X	X	X	X	X	X	X	X	X		X			13
1.3	X	X	X		X	X			X		X	X		X		X	10
2.1	X	X	X	X	X	X		X	X			X			X	X	11
2.2	X		X	X	X	X		X				X		X	X	X	10
2.3				X				X							X	X	4
3.1	X	X	X	X		X	X			X			X				8
3.2	X	X	X			X	X		X		X		X				8
3.3	X						X	X					X				4
4.1	X	X	X						X	X	X		X	X	X		9
4.2	X	X	X	X	X			X	X	X							8
5.1	X	X			X		X			X	X				X		7
5.2				X	X		X			X			X				5
	11	9	9	8	8	7	7	7	7	7	6	5	5	5	5	4	
Total subthemes per expert																	

## Discussion

This project aimed to identify the challenges in New York State from the perspective of leaders in various capacities throughout the State and address these challenges through research and funding of pilot projects. Experts identified stigma as a global issue and barrier to care for people who use substances. The World Health Organization defines stigma as “a mark of shame, disgrace, or disapproval that results in an individual being rejected, discriminated against, and excluded from participating in a number of different areas of society” [7]. Structural stigma refers to stigma that is codified in rules, policies, and/or practices within the health care industry [8]. Health system level stigma can result in patients avoiding treatment, communication barriers between patients and providers, and challenges in therapeutic alliances [8–10]. Additionally, individuals with substance use disorders (SUD) may internalize stigma from structural and societal experiences. Internalized stigma, or self-stigma, can be defined as “shame, evaluative thoughts, and fear of enacted stigma that results from individuals’ identification with a stigmatized group that serves as a barrier to the pursuit of valued life goals” [10]. This stigma can have deleterious effects for patients with SUD, resulting in delays in treatment, low self-esteem or low self-efficacy, and a lower quality of life, [11–13] and can serve as a deterrent to accessing substance use disorder treatment services, for example, problems with treatment compliance [14], withdrawal from social support networks [15], and loss of housing [16]. Additionally, people with SUDs may avoid treatment because of discrimination or fear of being rejected by friends or family who may negatively view their substance use [11, 12, 16].

Given that individuals with SUD often also have histories of trauma [16], it is important to acknowledge that re-traumatization that can occur alongside structural and internalized stigma [17]. Different regulatory structures for mental health and substance use disorder can lead to complexities in the way in which care is provided, cause barriers to accessing care, increase frustration with service provision, and reduce care quality. This can be due to conflicting policies, burdensome or confusing payment mechanisms, poor communication from siloed information systems, and difficult-to-access facility location [18, 19]. In New York, mental health services are regulated by the Office of Mental Health and substance use disorder services are regulated by the Office of Addiction Services and Supports. Providers are often forced to choose between primary mental health or substance use disorder to initiate care, when patients with co-occurring diagnoses should have both disorders treated simultaneously [19]. There are also fragmentation issues related to specialty training. Mental health training programs often do not include sufficient education on substance use disorders or co-occurring disorders [19]. The Substance Abuse and Mental Health Services Administration estimates that 80% of patients who receive care in mental health and substance use disorder systems do not receive the comprehensive services needed due to fragmentation [20], indicating that service fragmentation is an issue across States.

In a 2022 report to Congress, the United States Government Accountability Office (GAO) reported that one of the most significant barriers to accessing substance use disorder treatment is the shortage of qualified behavioral health providers [21]. This is particularly true in rural areas. The GAO estimates that 50% of all counties in the United States do not have access to a psychiatrist or addiction medicine counselor [21]. In 2023, the Health Resources and Services Administration designated more than 6,546 mental health provider shortage areas across the United States, meaning over 163 million people live without sufficient access to a mental health provider. To meet the need in these communities, the behavioral health workforce would need to grow by 8251 providers [22]. Ultimately, the GAO identified three main categories of barriers to the recruitment and retention of a skilled behavioral health workforce: financial, educational, and workplace. Financial refers to the reimbursement and compensation rates for behavioral health providers that impact the competitiveness of the field compared to other health professions. Educational barriers refer to the dearth of training available to new behavioral health providers, and the lack of a recruitment pipeline particularly as it relates to diverse and underserved populations. Workplace barriers refer to a paucity of licensed supervisors needed to train incoming providers, few funded internships, and provider burnout due to workload demands, particularly in the face of a growing opioid epidemic [22].

Extensive research has been conducted in each of these categories. In terms of competitive pay, literature suggests wages, benefits, and caseloads as substantial factors related to skilled workforce retention [23, 24]. Even in terms of health care specialties, pay in addiction medicine services has historically been lower compared to specialties that required similar training and education [25]. Beyond pay, Oser and colleagues found that treating clients with multiple psychosocial needs, high caseloads, and a heavy administrative burden are frequent causes of substance use disorder counselor burnout [26].

Turnover rates in substance use disorder agencies can be as high as 33%, resulting in high organizational costs related to recruitment and training, increased caseloads for existing staff, and delays in care [23]. Staff turnover can be due to many factors including low pay, administrative burden, and lack of support or supervision [23, 27]. Additionally, few clinicians receive training in addiction treatment given that most psychiatric programs do not provide training in addiction medicine or treatment of patients with co-occurring substance use disorder and mental health disorders [28, 29]. A 2015 study found that fewer than half of undergraduate social work programs have coursework on substance use disorders [30]. Training and supervision post-graduation is needed, with clear regulatory recommendations for recertification and scope of practice [31, 32].

Several barriers to accessing the continuum of services in substance use disorder prevention, treatment, and harm reduction exist. A continuous system would have a “no wrong door” approach for entry into needed services, shared resources and best practices, and a unified healthcare record system across institutions and agencies. The shared healthcare record system would facilitate reaching patients and monitoring individual progress, as well as assessing system effectiveness with long-term outcomes. Experts indicated that Title 42 may be a barrier to implementing this system.

In addition to stigma, accessibility to services, and a lack of skilled professionals in the workforce, there are challenges such as the persistent pipeline of illicit substances into the United States, provider hesitancy to provide medications for addiction treatment, and overarching social determinants of health. major In 2022, the Biden Administration allocated almost $6 billion in interdiction strategies to combat this pipeline, particularly with respect to fentanyl importation [21]. The administration has also increased funding to increase access to medications for opioid use disorder.

Additional barriers to expanding access to substance use disorder (SUD) care include provider attitudes and training. Providers can harbor negative sentiments about their patients with SUD, which can affect their willingness to prescribe them FDA-approved medications [9, 16, 33]. Discomfort in working with patients with SUD is also frequently cited, mainly due to lack of training and/or exposure to individuals with SUD [9, 16]. Additionally, there are many policy barriers to providers prescribing FDA-approved medications for SUDs such as training, institutional support, regulatory and licensing issues, and low reimbursement rates [34].

Finally, several social determinants also impact patients with SUD from accessing the continuum of care. They include structural barriers related to race/ethnicity, lower socio-economic status, language barriers, transportation, and lack of stable housing [35–37]. Moreover, pregnant patients and patients with children who have SUD diagnoses face unique challenges including a limited number of programs that allow children onsite, can accompany providers’ comfort working with pregnant patients, fear of becoming justice-involved, and potentially losing custody of their children [38, 39]. These barriers to evidence-based treatments is a critical issue that must be addressed expeditiously.

The results of this project were shared with the New York State Office of Addiction Services and Supports (OASAS) and were used to guide funding priorities for several pilot intervention and evaluation programs designed to make a practical impact on the issues raised. Successful outcomes in these projects will encourage dissemination and replications at larger scales to ameliorate the identified challenges.

### Limitations and recommendations for future research

Our sample of experts is based in one U.S. State. Conditions and issues may vary in other areas, especially internationally. Nearly all the experts interviewed are in organizational leadership positions; the perspectives of direct service staff may differ. It would be valuable to conduct similar research projects with these other sets of stakeholders. Substance use disorder service recipients may have different perspectives than service providers. Interviews with people who used opioids documented similarities in concerns regarding stigma and treatment requirements [40].

## Conclusions

A diverse group of experts identified siloed and fragmented systems, the need for workforce training, attitudes towards addiction, limitations in treatment access, and conditions in the current drug environment as the five major challenges to the optimal implementation of substance use disorder prevention, harm reduction, and treatment strategies. Despite the open-ended nature of the question regarding the greatest challenge, experts were remarkably consistent in the identification of major themes. Research, educational, and intervention agendas should focus on these topics as a means of improving the lives of patients at risk for or suffering from substance use disorders. Policies and practices should be informed by empirical research. This project helped to shape funding priorities; thus, we consider this project to be a successful application of science.

## Data Availability

Materials will be available upon reasonable request. Raw verbatim data will not be available for ethical reasons.

## Abbreviations

GAO: United States Government Accountability Office
MAT: Medication-assisted treatment
OASAS: New York State Office of Addiction Services and Supports
OUD: Opioid use disorders
SUD: Substance use disorders
